# Endothelin-1 gene polymorphisms and diabetic kidney disease in patients with type 2 diabetes mellitus

**DOI:** 10.1186/s13098-015-0093-5

**Published:** 2015-11-19

**Authors:** Claudete M. Zanatta, Daisy Crispim, Denise A. Sortica, Lucas P. Klassmann, Jorge L. Gross, Fernando Gerchman, Luís H. Canani

**Affiliations:** Federal University of Rio Grande do Sul, Porto Alegre, Brazil; Endocrine Division, Hospital de Clínicas de Porto Alegre, Federal University of Rio Grande do Sul, Porto Alegre, RS Brazil

**Keywords:** Endothelin-1, Diabetic kidney disease, Diabetes mellitus type 2

## Abstract

**Background and aims:**

Diabetic kidney disease (DKD) is the leading cause of end stage renal disease worldwide and is associated with increased cardiovascular mortality. The endothelin system has been implicated in the pathogenesis of arterial hypertension and renal dysfunction. In the present study, the association of DKD with polymorphisms in ET-1 (*EDN1*) and ETRA (*EDNRA*) genes was analyzed in patients with type 2 diabetes mellitus (T2DM).

**Methods:**

A case–control study was conducted in 548 white T2DM patients. Patients with proteinuria or on dialysis were considered cases and patients with normoalbuminuria were considered controls. Two polymorphisms in the *EDN1* gene (rs1800541 and rs57072783) and five in *EDNRA* gene (rs6842241; rs4835083; rs4639051; rs5333 and rs5343) were genotyped and haplotype analyses were performed.

**Results:**

The presence of rs57072783 T allele (TT/TG vs. GG) or rs1800541 G allele (GG/GT vs. TT) protected against DKD (OR = 0.69, 95 % CI 0.48–0.99, P = 0.049; and OR = 0.60, 95 % CI 0.41–0.88, P = 0.009, respectively). However in multivariate analyses, only the rs1800541 G allele remained independently associated with DKD (P = 0.046).

**Conclusions:**

The present study shows that ET-1 could be involved in the pathogenesis of DKD in patients with T2DM.

## Background

Diabetic kidney disease (DKD) is the leading cause of chronic kidney disease in patients starting renal replacement therapy [1] and it is associated with increased cardiovascular mortality [2]. Among patients starting renal replacement therapy, the incidence of DKD doubled over the 1991–2001 period. Fortunately, the number of new cases of end stage renal disease (ESRD) in people with diabetes or high blood pressure declined by about 2 percent in 2011 compared with 2010—the first decrease in more than 30 years—which may mean that people with those diseases are getting better treatments [1]. However, implementation of these measures remains far below desirable goals [3].

Endothelin-1 (ET-1) is the predominant isoform of the endothelin peptide family. It acts through the receptors type A (ETRA) and B (ETRB) [4, 5], leading to cell proliferation and vasoconstriction. Human studies and animal experiments have documented that renal synthesis of ET-1 is increased in chronic kidney disease [6, 7]. Endothelial dysfunction increases ET-1 production, leading to vascular hypertrophy, atherogenesis and, in the kidney, glomerulosclerosis [8–10]. Elevated plasma ET-1 levels have been reported in patients with diabetes mellitus [11–13]. We have previously demonstrated that plasma ET-1 levels increases as the urinary albumin excretion increases [14], and that the human kidney with DKD overexpress ET-1 and ETRA [15].

However, it stills not clear whether ET-1 is involved in the pathogenesis of DKD or it is only a secondary modulator that promotes progression after disease onset. In this context, genetic studies could be helpful in distinguish primary disease effects from secondary ones. The aim of this study was to evaluate the association between genetic variants of the ET-1 and ETRA genes (*EDN1* and *EDNRA*) and DKD in patients with type 2 diabetes mellitus (T2DM).

## Subjects and methods

### Subjects

The sample of this nested case–control study is composed of 548 unrelated white patients with T2DM who were included in a multicenter study that started recruiting patients in 2002 [16]. The original project was designed to assess risk factors for the chronic complications of T2DM, and includes four centers located at general hospitals in the state of Rio Grande do Sul, Brazil: Grupo Hospitalar Nossa Senhora da Conceição, Hospital São Vicente de Paula, Hospital Universitário de Rio Grande, and Hospital de Clínicas de Porto Alegre. Ethnic groups were defined on the basis of self-classification. Only patients who described themselves as white were included. In the state of Rio Grande do Sul, white persons are mainly of European ancestry (mostly Portuguese, Spanish, Italian, and German descent).

Patients underwent a clinical and laboratory evaluation as previously described [14]. Blood pressure (BP) was measured in the sitting position twice, with a 5-min interval between measurements. Weight and height were used to calculate body mass index (kg/m^2^). Cases were defined by the presence of macroalbuminuria (proteinuria) or dialysis treatment. Albumin excretion rate (AER) was measured in at least three urine collections. Patients were classified in accordance with previously defined local standards [17] as normoalbuminuric (AER <20 μg/min or <17 mg/L on a 24-h timed urine or spot random sterile urine sample respectively) or macroalbuminuric (AER >200 μg/min or >174 mg/L or current dialysis). T2DM patients with 5 years or more of disease and normoalbuminuria formed the control group. Et-1 was measured in a subgroup of subjects (n = 111) regardless being in the control or case group. Since many factors can affect plasma ET-1 levels, ET-1 was measured after discontinuation of angiotensin-converting enzyme (ACE) inhibitor therapy for at least 2 weeks. For this subgroup, a more stringent exclusion criteria was applied and include: renal impairment (serum creatinine ≥ 1.5 mg/dL), any cardiovascular event during the 6 months preceding enrolment (stroke, myocardial infarction, unstable angina, lower limb amputation, bypass surgery, or percutaneous coronary intervention), heart failure (New York Heart Association class II or worse), liver disease (history of liver disease or elevated liver enzymes), any infectious, inflammatory or malignant process. Mean plasma ET-1 values were analyzed according to different genotypes of the *EDN1* and *EDNRA* polymorphisms.

The information obtained from the study did not influence patient diagnosis or treatment in any way. The research ethics committees of the participating centers approved the study protocol, and all patients provided written informed consent.

### Laboratory methods

Laboratory analyses were performed in serum blood sample collected after a 12-h fast as previously described [14]. Fasting blood glucose was determined by the glucose oxidase method; plasma triglycerides and cholesterol, by enzymatic methods; AER, by immunoturbidimetry (Sera-Pak immuno microalbuminuria, Bayer, Tarrytown, NY, USA; mean intra- and interassay coefficients of variance of 4.5 and 7.6 %, respectively); serum creatinine by Jaffe’s reaction; glycated hemoglobin (HbA_1c_) by ion-exchange HPLC (Merck-Hitachi L-9100 GhB Analyser, reference range 4.7–6.0 %). Plasma ET-1 was measured by ELISA as previously described [14].

### Genotyping

Genomic DNA was extracted from blood leukocytes by a salting-out procedure [18]. Evaluation of the polymorphism rs4639051 in intron 3 of the *EDNRA* gene was done by digesting polymerase chain reaction (PCR) products with the *Hha*I restriction enzyme (New England Biolabs, Inc., Ipswich, MA, USA). Digestion fragments were resolved on 2 % agarose gels containing ethidium bromide and visualized under ultraviolet illumination. Genotypes of the rs4639051 polymorphism were recorded using the ImageMaster VDS system (GE HealthCare, London, UK). The primer sequences used for this polymorphism are forward 5′-GAC TAT CCC AGA CCA CAC CTT CA-3′ and reverse 5′-GCT CAG GGC TGC CAA CTC C-3′.

Genotyping of the rs4835083 (G/A; intron 1), rs1568136 (T/A; intron 2), rs5333 (C/T; exon 6) and rs5343 (T/C; exon 8) polymorphisms in the *EDNRA* gene and rs1800541 (-T1370G; promoter region) and rs57072783G/T (*Lys*198*Asn*; exon 5) in the *EDN1* gene was performed using specific primers and probes (Custom TaqMan Genotyping Assay 40×—Life Technologies, Foster City, CA, USA). One allele-specific probe was labeled with VIC dye and the other was labeled with FAM dye. The total reaction volume of 5 μL included 2 ng of genomic DNA, TaqMan Genotyping Master Mix 1× (Life Technologies), and Custom TaqMan Genotyping Assay 1× specific for each polymorphism. Plates were then placed in a real-time PCR thermal cycler (7500 Fast Real PCR System; Life Technologies) for 10 min at 95 °C, followed by 40–50 cycles at 95 °C for 15 s and at 63 °C for 60 s. Fluorescence data files from each plate were analyzed using automated allele-calling software (SDS 2.1; Life Technologies). The lowest genotyping success rate was obtained for the rs5333 polymorphism (95 %) among controls and for the rs5333 and rs4639051 polymorphisms (95 %) among cases.

*EDNRA* gene polymorphisms were selected from the International HapMap Project [19]. Due to linkage disequilibrium between some of the 58 common polymorphisms, at least five polymorphisms had to be genotyped to estimate all haplotypes with more than 5 % frequency and that would cover more than 90 % of all possible *EDNRA* gene polymorphisms haplotypes. The *EDN1* rs1800541 and rs57072783 polymorphisms were selected on the basis of a previous study [20], which reported that these two polymorphisms are in almost complete linkage disequilibrium with other polymorphisms in this gene, thus covering more than 90 % of gene variability.

### Statistical analysis

Allele frequencies were determined by gene counting. The Chi square test was used to verify the Hardy–Weinberg equilibrium (HWE) and compare genotype and allele frequencies. Genotypes were evaluated assuming different genetic models, including additive, recessive and dominant. We examined widely used measures of linkage disequilibrium (LD), Lewontin’s D′ |D′| and *r*^2^ [21], between all pairs of biallelic loci in the *EDNRA* or *EDN1* genes. Phase 2.1 program was used to infer the haplotypes derived from the combination of the *EDNRA* and *EDN1* gene polymorphisms [21]. This method is based in a Bayesian statistical method [21]. Phase 2.1 was also used to compare the distributions of different *EDNRA* and *EDN1* gene haplotypes between cases and controls through permutation analyses of 1000 random replicates [22].

The clinical and laboratory comparisons between groups were performed by the unpaired Student’s test or the Chi square test as appropriate. Continuous variables were expressed as means and standard deviations (SD). Variables with a skewed distribution (serum creatinine, albuminuria, triglycerides, and ET-1) were logarithmically transformed and were presented as median (interquartile range). Odds ratio (OR) was used to assess the magnitude of the association between different genotypes and DKD with 95 % confidence intervals (95 % CI). Bonferroni’s test was used to correct for multiple comparisons. Multivariate logistic regression analyses were carried out to control for possible confounding factors and to assess the independence of associations between genotypes and DKD. A two-tailed P value of <0.05 was considered statistically significant. All statistical analyses were performed in the SPSS—Windows 16.0 environment.

## Results

### Sample profile

Table 1 presents the main clinical features of patients according to renal status. Cases were more often males and had a longer duration of T2DM than controls. Cases also had lower HDL cholesterol levels and higher triglycerides and blood pressure values as compared with controls. By definition, serum creatinine was higher among cases as compared with controls. These differences held after Bonferroni’s correction for multiple comparisons.

**Table 1:** Clinical and laboratory profile of patients with type 2 diabetes mellitus according to renal status

	Controls (n = 308)	Cases (n = 240)	P*
Male gender, n (%)	118 (38.3)	144 (60.0)	<0.001
Duration of diabetes (years)	13.4 ± 7.2	17.2 ± 9.6	<0.001
Age (years)	60.6 ± 9.7	61.2 ± 9.9	0.477
BMI (kg/m^2^)	28.4 ± 4.7	28.4 ± 5.2	0.999
Systolic blood pressure (mmHg)	142 ± 23.5	149 ± 24.1	<0.001
Diastolic blood pressure (mmHg)	85 ± 13.5	86 ± 14.0	0.398
HbA1c (%)	6.88 ± 1.57	6.85 ± 2.06	0.847
Fasting plasma glucose (mg/dL)	161.8 ± 59.5	168.3 ± 75.3	0.260
Serum creatinine (mg/dL)	0.9 (0.5–1.4)	2.9 (0.6–13.9)	<0.001
Cholesterol, total (mg/dL)	206 ± 44.2	203.1 ± 52.6	0.469
Cholesterol, HDL (mg/dL)	46.7 ± 11.5	41.7 ± 11.7	<0.001
Triglycerides (mg/dL)	143 (40–659)	171 (45–1265)	<0.001

Data expressed as mean ± SD, median (range) or %

* P values computed by Chi square or Student’s *t* test as appropriate. Bonferroni threshold for multiple comparisons = 0.0042

### Genotype and allele distributions

The distributions of the *EDN1* and *EDNRA* gene polymorphisms in T2DM patients with and without DKD are shown in Table 2. All genotypes were in Hardy–Weinberg equilibrium (P > 0.05; data not shown). The *EDN1* rs1800541 polymorphism is in moderate LD with the *EDN1* rs57072783 polymorphism (|D′| = 0.823 and *r*^2^ = 0.788). LD coefficients (|D′| and *r*^2^) between all biallelic combinations of the five *EDNRA* gene polymorphisms are presented in Table 3. The analyzed *EDNRA* gene polymorphisms are not in significant LD with each other.

**Table 2:** Genotypic and allelic frequencies of *EDN1* and *EDNRA* polymorphisms in white type 2 diabetic patients according to renal status

	Genotype frequencies		P*	OR (95 % CI)		Allele frequencies		P**
	Controls	Cases				Controls	Cases	
*EDN1*								
rs1800541	n = 304	n = 239						
TT	200 (65.8)	182 (76.2)	0.031	1	T	0.806	0.866	0.011
GT	90 (29.6)	50 (20.9)		0.61 (0.41–0.91)	G	0.194	0.134	
GG	14 (4.6)	7 (2.9)		0.55 (0.22–1.39)				
rs57072783	n = 289	n = 224						
GG	169 (58.5)	150 (67.0)	0.050	1	T	0.234	0.183	0.062
TG	105 (36.3)	66 (29.5)		0.71 (0.49–1.03)	G	0.766	0.817	
TT	15 (5.2)	8 (3.6)		0.60 (0.25–1.46)				
*EDNRA*								
rs4835083	n = 300	n = 233						
AA	115 (38.3)	100 (42.9)	0.513	1	A	0.622	0.644	0.498
AG	143 (47.7)	100 (43.9)		0.80 (0.56–1.16)	G	0.378	0.356	
GG	42 (14.0)	33 (14.2)		0.90 (0.53–1.53)				
rs1568136	n = 308	n = 240						
TT	149 (47.7)	111 (46.2)	0.852	1	A	0.307	0.323	0.254
AT	129 (42.1)	103 (42.9)		1.07 (0.75–1.53)	T	0.693	0.677	
AA	30 (10.1)	26 (10.8)		1.16 (0.65–2.08)				
rs4639051	n = 287	n = 230						
AA	183 (63.4)	157 (68.3)	0.040	1	A	0.814	0.826	0.113
AG	103 (35.9)	66 (28.7)		0.75 (0.51–1.09)	G	0.186	0.174	
GG	2 (0.7)	7 (3.0)		4.08 (0.84–19.29)				
rs5333	n = 284	n = 230						
TT	137 (48.2)	125 (54.3)	0.373	1	T	0.695	0.728	0.282
TC	121 (42.6)	85 (37.0)		0.77 (0.53–1.11)	C	0.305	0.272	
CC	26 (9.2)	20 (8.7)		0.84 (0.45–1.59)				
rs5343	n = 299	n = 235						
CC	120 (40.1)	105 (43.8)	0.470	1	C	0.698	0.645	0.113
CT	38 (46.2)	97 (40.9)		2.92 (1.85–4.61)	T	0.302	0.355	
TT	41 (13.7)	36 (15.3)		1.00 (0.60–1.69)				

Genotype frequencies expressed as n (%) and allele frequencies shown as proportions

Bonferroni threshold for multiple comparisons = 0.0071

*OR (95* *% CI)* odds ratio (95 % confidence interval)

* P values computed by Chi square test for genotypes comparisons

** P values computed by Chi square test for allele frequencies comparisons

**Table 3:** Linkage disequilibrium (|D′| and *r* ^2^) between all biallelic loci of the *EDNRA* gene

	|D′|					
*r* ^2^		rs4835083	rs1568136	rs4639051	rs5333	rs5343
	rs4835083	–	0.576	0.133	0.359	0.452
	rs1568136	0.260	–	0.452	0.665	0.579
	rs4639051	0.007	0.099	–	0.802	0.635
	rs5333	0.090	0.394	0.349	–	0.657
	rs5343	0.182	0.087	0.051	0.100	–

Cases had a lower frequency of the *EDN1* rs1800541 G/G and G/T genotypes compared to controls (P = 0.031). Assuming a dominant model of inheritance (GG/GT *vs*. TT), presence of the G allele was associated with an OR of 0.60 (95 % CI 0.41–0.88, P = 0.009). G allele frequency was 0.194 in controls versus 0.134 in cases (P = 0.01). Similar results were obtained when controls with more than 10 years of T2DM were analyzed (data not shown). Being conservative, both genotype and allele frequencies of rs1800514 polymorphism were not statistically different between case and control subjects after Bonferroni’s correction (Table 2). However, on multivariate analysis, presence of the rs1800541 G allele remained independently associated with lower frequency of DKD after controlling for gender, T2DM duration, systolic BP, HbA1c and HDL cholesterol (OR = 0.67, 95 % CI 0.42–0.89; P = 0.046). It bears noting that the prevalence of DKD was not different when assuming a recessive model of inheritance for the G allele (GG vs. GT/TT; data not shown).

The *EDN1* rs57072783 T/T and T/G genotypes exhibited a borderline association with DKD (P = 0.05). Assuming a dominant model of inheritance, presence of the T allele (TT/TG vs. GG) was associated with an OR of 0.69 (95 % CI 0.48–0.99, P = 0.049), but this significance was not significant after multiple logistic regression analysis adjusting for gender, T2DM duration, systolic BP, HbA1c and HDL cholesterol (P = 0.196). The prevalence of DKD was not significantly different when assuming a recessive model of inheritance for the T allele (TT vs. TG/GG; data not shown).

Genotype and allele frequencies of the *EDNRA* rs4835083, rs1568136, rs5333 and rs5343 polymorphisms were similar among cases and controls (all P values >0.10). The genotype distribution of the *EDNRA* rs4639051 polymorphism was significantly different between cases and controls (P = 0.04), but this association was lost after Bonferroni’s correction.

The frequency of the heterozygous genotype was slightly lower and that of the major genotype (A/A) slightly higher among cases. However, given that the G allele is rare, the genotype distribution pattern of this polymorphism could not be characterized as additive, dominant or recessive. The allele frequencies of the rs4639051 polymorphism were not different between cases and controls (P = 0.113). On multivariate analysis, no association was observed between the rs4639051 polymorphism and DKD after controlling for gender, systolic BP, T2DM duration, HbA1c and HDL cholesterol (P = 0.057).

### Haplotype distributions

A Bayesian statistical method was used to estimate the frequencies of different haplotypes produced by the combination of the *EDN1* or *EDNRA* gene polymorphisms. All four expected haplotypes constructed by the combination of the two *EDN1* polymorphisms were observed (Table 4). The five polymorphisms of the *EDNRA* gene result in 24 different haplotypes, but only those with a frequency of >5 % are presented in Table 4. For both genes, permutation analyses showed that haplotype distributions were not statistically different between case and control subjects.

**Table 4:** *EDN1* and *EDNRA* haplotype frequencies in patients with type 2 diabetes mellitus according to renal status

Haplotype frequencies	Controls	Cases	P*
*EDN1*	n = 311	n = 188	
T/G	0.752	0.749	0.424
T/T	0.059	0.058	
G/G	0.053	0.044	
G/T	0.136	0.149	
*EDNRA*	n = 242	n = 276	
G/A/A/C/C	0.089	0.072	0.278
G/A/G/C/C	0.057	0.050	
G/T/A/T/T	0.100	0.090	
A/T/A/T/C	0.273	0.286	
A/T/A/T/T	0.178	0.180	

The first letter of the *END1* haplotype refers to the rs1800541 polymorphism and the second to the rs57072783 polymorphism. The first letter of the *EDNRA* haplotype refers to the rs4835083 polymorphism, the second to the rs1568136, the third to the rs4639051, the fourth to the 5333 and the last to the rs5343 polymorphism

*n* number of chromosomes

* P values for comparison of haplotype frequencies between patients with or without diabetic kidney disease were calculated using permutation tests (1000 replications)

### ET-1 levels

ET-1 was measured in 111 patients who were able to discontinue medications that could interfere with ET-1 levels and did not have any acute or chronic conditions that could be associated with increased levels of ET-1. No differences in ET-1 levels were found among any of the polymorphisms analyzed (data not shown).

## Discussion

A previous study reported that patients with T2DM had elevated ET-1 levels as compared with nondiabetic subjects [11]. Furthermore, plasma ET-1 levels are higher in macroalbuminuric than normoalbuminuric patients [14]. ET-1 is the most powerful endogenous vasoconstrictor, and has profibrotic and proinflammatory effects [23]. It has been found to affect three different aspects of renal physiology: vascular and mesangial tone; sodium and water excretion; and cell proliferation and matrix formation [13, 24]. Immunohistochemical studies of kidney biopsies of subjects with DKD show overexpression of ET-1 and ETRA [15]. In experimental rat models, endothelin antagonist treatment has a nephroprotective effect, correcting both the initial hyperfiltration and its progression to clinical DKD [9, 10] and reducing inflammation and podocyte injury [25]. The therapeutic potential of endothelin receptor antagonists in human kidney diseases featuring chronic proteinuria (including DKD) was recently reviewed [26]. A recent double-blind, placebo-controlled trial showed that the endothelin antagonist avosentan significantly reduced albuminuria when added to standard treatment in T2DM patients [27].These studies provide further evidence of the role of ET-1 in the pathogenesis of DKD and of its status as a promising treatment target for this complication.

In the present study, two variants in the *EDN1* gene were associated with DKD protection in white patients with T2DM. Presence of the *EDN1* rs1800541 G allele and rs57072783 T allele was associated with a decreased risk of DKD. The association was stronger for the rs1800541 polymorphism and more evident when assuming a dominant model of inheritance. However, none of the polymorphisms were associated with plasma ET-1 levels.

A previous study investigating potential associations between a set of 45 polymorphisms located in 20 candidate genes and DKD in T2DM patients from the Czech Republic also reported a relationship between an *EDN1* gene polymorphism (8002 G/A) and this diabetic complication using multi-locus analysis (P = 0.033) [28]. However, this association did not remain statistically significant after adjusting for diabetes duration, HbA1c, diastolic BP and the presence of other DKD-associated polymorphisms located in three different genes, namely *AGER*-429T/C and 2184A/G, *LTA* 252A/G, and *NOS3* 774C/T and E298D. In T2DM obese subjects, the *EDN1* 8002T polymorphism was associated with a higher prevalence of combined microangiopathy (neuropathy/retinopathy/nephropathy) (P = 0.035) [29].

A number of previous studies have linked *EDN1* rs1800541 and rs57072783 polymorphisms with hypertension in individuals with overweight and obesity [30–32], as well as with HDL cholesterol metabolism [33]. These are known predisposing factors for the development of DKD. The relationship between these two polymorphisms and impaired renal function in a nondiabetic population was studied in the PREVEND Study cohort [20]. Haplotype analysis revealed that individuals carrying both the *EDN1* rs1800541 G allele and *EDN1* rs57072783 T allele showed decreased glomerular filtration and lower creatinine clearance than carriers of other haplotypes. No significant difference in ET-1 plasma levels was observed between haplotype groups. However, in this study, only normoalbuminuric and microalbuminuric patients were analyzed, whereas patients with overt proteinuria were excluded.

Recently, the rs57072783 T allele was associated with delayed onset of T2DM and reduced risk of diabetic retinopathy in a Chinese sample [34]. However, the rs57072783 T/T genotype was associated with elevated plasma ET-1 levels in pregnant women, and the T allele has been associated with raised systolic BP [35]. In our study, the protective association observed between presence of the rs57072783 T allele (TT or TG, dominant inheritance model) and DKD was statistically weak and did not remain independently associated with DKD after adjusting for gender, diabetes duration, systolic BP, HbA1c and HDL cholesterol.

Activation of ETRA in renal cells leads to a complex signaling cascade resulting in stimulation of mesangial cell proliferation, contraction, hypertrophy and extracellular matrix accumulation [4]. These renal alterations are associated with the onset and progress of DKD. Besides, ETRA is overexpressed in kidney of subjects with DKD [15]. SNP rs4639051 was the more promising polymorphism evaluate in EDNRA gene (P = 0.04). However, likewise rs57072783, the association did not remain after controlling for possible confounding factors in multivariate analysis. Since BP could be involved in the pathway of endothelin system leading to DKD, we performed the multivariate analysis excluding this parameter. In both cases, the main result did not change (data not shown). The fact that we were not able to show an association of rs57072783 and rs4639051 with DKD, does not exclude their potential role. This might be evaluated in a larger sample of subjects.

A lower ET-1 level would be expected with the protective genotypes of EDN1 gene polymorphisms (rs1800541 and rs57072783). However, no difference was found in the present study. Since many situations could interfere with ET-1 levels, ET-1 could be measured in only 111 selected subjects. Those in whom the genetic effect was likely to be more marked were excluded a priori. This would be the case for those with advanced renal disease.

On the other hand, Tanaka et al. [36] investigated expression of the rs57072783 polymorphism in ET-1 in vitro: rs57072783 T allele cells were transfected and compared with G allele transfected cells with preproET-1 in three different cell lines. The authors measured the levels of ET-1 and its precursor, big ET-1, in the culture supernatant and did not find a significant difference in levels of either substance between the T-type and G-type transfectant cells, suggesting that this polymorphism does not, in fact, play a significant role in ET-1 levels. Even though some minor effect on the processing of preproET-1 to mature ET-1 cannot be totally ruled out, it is more possible that rs57072783 is not the causal polymorphism, but only occurs in LD with an unknown functional polymorphism in the *EDN1* gene. The rs57072783 polymorphism leads to an amino acid change in ET-1 precursor, but not in mature ET-1. Therefore, it is also possible that this polymorphism might affect the protein structure, modulating its binding affinity, without any changes in ET-1 levels. To the best of our knowledge, no other study has evaluated ET-1 levels according to different genotypes of the rs1800541 polymorphism.

We also used a Bayesian statistical method to estimate the frequencies of different haplotypes constructed by combination of the *EDN1* or *EDNRA* gene polymorphisms. However, these haplotype analyses did not add any further information to the single polymorphism analyses, i.e. the frequencies of all observed *EDN1* and *EDNRA* gene haplotypes were not statistically different between case and control subjects. The selection of polymorphisms evaluated in our study sought to cover the most common haplotypes, and was based on a previous publication [20] and on HapMap Project data. Unfortunately, we cannot exclude the possibility that less frequent *EDN1* or *EDNRA* gene haplotypes could have some effect on development of DKD.

The limitations of this study include its cross-sectional design, which can only lead to conclusions about associations, not causality. Still on the topic of the cross-sectional design, 116 patients were on dialysis; therefore, we could expect changes in glycemic control, weight and HbA1c in this subset. This could explain our failure to find or decreasing the magnitude of differences in traditional risk factors between cases and controls. Therefore, we decided a priori which variables would be included in multivariate analysis rather than only using those with statistical significance on univariate analyses. Interestingly to note, the group of cases had more males, had longer diabetes duration, higher systolic BP and worst lipid profile. However, presence of the rs1800541 G allele remained associated with DKD after controlling for these possible confounding factors. Therefore, we believe these differences did not affect the main findings. Other factors could have interfered with our findings; for instance, the possibility of stratification bias cannot be discarded. Nevertheless, we only analyzed subjects who self-reported ethnicity as white, thus reducing the risk of false-positive or false-negative associations due to this bias. Therefore, the findings of the present study must be taken carefully taking into account that the reported associations did not held after Bonferroni’s correction.

In conclusion, the present study demonstrated an independent association between a genetic variant in the *EDN1* gene and DKD in white patients with T2DM. The effect appears minor, but is supported by the existing hypothesis that the ET-1 system would be involved in the development of DKD. Larger confirmatory studies in other populations are required to define the role of these polymorphisms in DKD.

## Abbreviations

DKD: diabetic kidney disease
ESRD: end stage renal disease
ET-1: endothelin-1
ETRA: receptors type A
ETRB: receptors type B
EDN1: genetic variants of the ET-1 genes
EDNRA: genetic variants of the ETRA genes
T2DM: type 2 diabetes mellitus
BP: blood pressure
AER: albumin excretion rate
ACE: angiotensin-converting enzyme
PCR: polymerase Chain Reaction
HWE: Hardy–Weinberg equilibrium
LD: linkage disequilibrium
OR: odds ratio
